# A Decoupled Fractional-Order Kalman Filter for Accelerometer Tilt Angle Estimation

**DOI:** 10.3390/s26165227

**Published:** 2026-08-18

**Authors:** Naiming Wu, Xu Liu, Houzeng Han, Jian Wang

**Affiliations:** 1 School of Geomatics and Urban Spatial Informatics, Beijing University of Civil Engineering and Architecture, Beijing 102616, China; 1108130024004@stu.bucea.edu.cn (N.W.); hanhouzeng@bucea.edu.cn (H.H.); wangjian@bucea.edu.cn (J.W.); 2 Beijing Key Laboratory of Precision Reconstruction and Health Monitoring for Architectural Heritage, Beijing 100044, China

**Keywords:** tilt angle estimation, accelerometer, Kalman filter (KF), decoupled fractional-order Kalman filter (DFKF)

## Abstract

Accelerometer-based tilt angle estimation is widely used in engineering monitoring, yet random noise and outliers degrade its accuracy. Integer-order Kalman filters suppress noise, but their Markovian model assumes that the current state alone is sufficient to predict the next, neglecting the influence of earlier states on slowly varying processes. Fractional-order Kalman filters incorporate historical states into the prediction. However, the conventional formulation shares a single transition matrix between the state and covariance predictions, underestimating the prediction uncertainty, while the memory mechanism propagates gross errors across iterations. To overcome these limitations, this paper proposes a decoupled fractional-order Kalman filter (DFKF). The method assigns independent transition matrices to the state and covariance predictions, where a scaling coefficient inflates the predicted covariance to lower the prediction weight and strengthen reliance on measurements. A front-end gross-error pre-elimination strategy combining second-order differencing with adaptive peak detection is further introduced to block outlier propagation at the source before it enters the memory mechanism. Simulations under varying noise levels and gross-error conditions show that DFKF achieves a mean RMSE (Root Mean Square Error) of 0.147°, representing reductions of 17.4% and 6.4% over KF (0.178°) and FKF (0.157°), respectively, and a mean MaxAE (Maximum Absolute Error) of 0.548°, outperforming KF and FKF by 24.5% and 10.3%. Under gross-error conditions, DFKF converges in 0.012 s on average, approximately 2.8 times faster than KF and FKF, and the pre-elimination strategy restores accuracy to near-error-free levels.

## 1. Introduction

In the past decade, tilt angle estimation has been widely used in various fields, including smart wearable devices, robotics, vehicle control, and high-rise infrastructure monitoring, often proving to be more practical than full 3D orientation estimation in many applications. Accelerometers serve as the primary tilt angle estimation devices, and tilt angle estimation typically involves a two-step process consisting of initial coarse estimation and subsequent fine estimation. The coarse estimation determines roll and pitch angles by analysing the vector relationship between the measured acceleration and the local gravitational field. The fine estimation then applies filtering techniques to enhance the accuracy of these initial measurements.

The Kalman filter (KF) and its variants are among the most widely used filtering techniques for tilt angle estimation using accelerometers. For instance, Weng et al. [1] proposed a wireless tilt angle estimation system based on multiple accelerometers, though the algorithm relied solely on the standard KF for noise reduction. Briales et al. [2] introduced a fusion method combining an extended Kalman filter (EKF) with a gradient descent algorithm (GDA), integrating MEMS accelerometers, gyroscopes, and geometric compensation to mitigate the impact of translational acceleration on tilt angle estimation, thereby achieving high-precision results. Ding et al. [3] developed an error-state Kalman filter (ESKF)-based data fusion algorithm, incorporating MEMS accelerometer and magnetometer data to enable high-accuracy, continuous 3D attitude estimation in both dynamic and static environments. Further advancements include the work of Hajiyev [4], who proposed a singular value decomposition (SVD)-assisted EKF algorithm. By preprocessing magnetometer and sun sensor data to linearize measurement inputs and fusing accelerometer and gyroscope data, this method simultaneously estimated satellite attitude angles and angular velocities, enhancing the reliability of tilt and motion parameter estimation for small satellites under complex operating conditions. Deibe et al. [5] introduced a time-varying Kalman filter (TVKF)-based low-acceleration adaptive attitude estimator, which employed a parameter *β* to switch between dynamic and static models. Experimental results demonstrated its superiority over ten benchmark algorithms, achieving an optimal balance between high dynamic adaptability and low computational cost. Beyond conventional Kalman variants, Akbari et al. [6] employed a linear-parameter-varying (LPV) H∞ filter to estimate roll and pitch from IMU data, reducing the filter’s sensitivity to inaccurate noise models and initial conditions. Anvari et al. [7] proposed a real-time adaptive Kalman filter whose forgetting factor is computed online from recent estimation errors, achieving substantial reductions in roll and pitch RMS error under dynamic excitation. Lan et al. [8] fused the data of a MEMS-IMU array and employed an adaptive Kalman filter that estimates the measurement noise online to adjust the filter gain for non-stationary motion attitude measurement. Most recently, Wei et al. [9] presented a robust adaptive attitude estimation algorithm (RAESKF) based on ESKF, incorporating direction cosine matrix (DCM) decomposition and online noise covariance adjustment. This approach improved pitch/roll angle accuracy by up to 67.21%. More recently, Chen et al. [10] proposed a robust Kalman filter that adaptively adjusts the measurement-noise covariance from the filtering innovations to compensate for external accelerations, markedly improving the pitch estimation accuracy of low-dynamic vehicles.

Analysis of these developments reveals two dominant research trends in tilt angle estimation. The first focuses on improving accuracy through multi-sensor fusion (gyroscopes, magnetometers), though this often necessitates expensive high-precision gyroscopes, limiting cost-effectiveness for commercial applications. In contrast, the second trend leverages algorithmic enhancements to KF-based methods, enabling comparable accuracy using low-cost accelerometers alone—a more scalable solution for widespread deployment. However, more sophisticated variants of the Kalman filter inevitably entail higher computational costs, while the relationships among filter design parameters (e.g., noise covariances ***Q*** and ***R***, or state dimensions) become increasingly intricate. Some originally independent model parameters, when forced into the same filtering framework, may lead to model mismatch, ultimately degrading estimation accuracy and increasing computational burden. This issue can be addressed through decoupling.

From the perspective of system classes, the approaches reviewed above are mainly formulated for linear or weakly nonlinear discrete-time stochastic systems, in which the tilt (attitude) dynamics evolve slowly relative to the sampling rate and the sensor noise can be approximated as zero-mean Gaussian white noise. Systems of this type are frequently encountered in industrial practice, and the corresponding filtering techniques have been successfully applied to the structural health monitoring of high-rise buildings, bridges and transmission towers, deformation and landslide monitoring [11], geotechnical subsidence monitoring, wind-turbine tower inclination measurement, the attitude levelling of construction and agricultural machinery, robotic and self-balancing platforms, and consumer wearable devices [12]. In such applications, low-cost MEMS accelerometers are typically deployed as long-term, unattended sensing nodes, so the estimation algorithm must simultaneously provide high accuracy, robustness against outliers and low computational cost, which is exactly the design objective of the present work.

The decoupled Kalman filter (DKF) refers to the process of isolating interdependent components or parameters in the Kalman filter, allowing them to operate independently to improve estimation precision or computational efficiency. For instance, Gao et al. [13] proposed a distributed decoupled Kalman filter based on singular value decomposition (SVD), which decomposes the original system equation into two reduced-order subsystems and constructs a diagonal weighting matrix to enable independent state estimation while enhancing computational efficiency. Similarly, Li et al. [14] introduced a decoupled adaptive Kalman filtering method for signal denoising, where Allan variance analysis was employed to estimate the measurement noise variance. By ensuring complete independence between the Kalman filtering process and noise variance estimation, mutual coupling between the filter and noise estimator was avoided, resulting in superior denoising performance. Marelli et al. [15] developed a distributed decoupled Kalman filtering approach, where the global Kalman estimate is decomposed into a linear combination of multiple independent local filters. This method ensures that each local filter relies solely on local measurements and global parameters, unaffected by propagation errors from the full observation vector, thereby improving estimation accuracy. Yi et al. [16] proposed a decoupled direction cosine matrix (DCM) estimator within EKF and UKF frameworks, isolating pitch/roll components from magnetometer data by hierarchically fusing accelerometer and magnetometer measurements. Rong et al. [17] designed an EKF-based attitude estimator that introduces a diagonalizable regulation matrix into the update-stage gain to eliminate the effect of magnetometer measurements on the pitch and roll angles. Li et al. [18] introduced a partially adaptive decoupled EKF (PADEKF) for attitude estimation using dual independent quaternions, decoupling attitude and heading to eliminate magnetic noise interference. More recently, Jung et al. [19] formalized an isolated Kalman filtering framework that designs decoupled estimators for systems with sparsely coupled outputs while preserving consistency with the centralized estimator. Collectively, these studies demonstrate that while these algorithms all employ decoupling, their methods, targets, and objectives differ. Some decouple observation vectors to prevent error propagation, while others isolate output parameters to avoid mutual interference. Nevertheless, their core principle remains consistent: eliminating coupling between specific Kalman filter elements to enhance either precision or computational performance. However, the decoupled Kalman filter only addresses errors arising from coupled model design parameters without directly eliminating algorithmic model errors. It is well established that conventional Kalman filters achieve computational tractability through state model linearization. This linearization process inevitably introduces accuracy loss (due to discarded higher-order terms), creating an inherent discrepancy between the linearized model and the true system dynamics.

The fractional-order Kalman filter (FKF) provides an alternative approach that enables more accurate state estimation through fractional-order calculus, which exhibits unique “memory” properties. Unlike conventional methods, FKF preserves the original physical system representation in its state equations without accuracy compromise. While computational intensity initially limited its applications, modern computing capabilities have enabled successful implementations across diverse fields including electronics, viscoelastic materials, bioengineering, robotics, signal processing, control systems, and navigation. Recent advances demonstrate FKF’s growing potential: Sierociuk et al. [20] developed enhanced FKF formulations for networked systems with packet losses. Xiong et al. [21] achieved superior accuracy and physical fidelity by integrating fractional calculus with Butler–Volmer equations through constant phase elements. Ha et al. [22] implemented an extended fractional-order Kalman filter (EFKF) for indoor air quality monitoring, while Chen et al. [23] developed a fractional-order composite circuit model for highly robust state-of-energy estimation. Chai et al. [24] proposed an adaptive fractional-order cubature Kalman filter with initial-value compensation for lithium-ion battery state-of-charge estimation, and Rodríguez-Iturriaga et al. [25] introduced a dual fractional-order EKF that jointly estimates the state of charge and the fractional parameters of the battery model. Zhao et al. [26] further advanced the field through a robust fractional-order EKF (RFEKF) that significantly improves GNSS navigation and positioning accuracy. More recently, robust and adaptive designs have been incorporated into the fractional-order framework: Nosrati et al. [27] derived a robust fractional-order singular Kalman filter for uncertain discrete-time systems with proven convergence, and Mu et al. [28] developed an adaptive robust fractional-gain interpolatory cubature Kalman filter for nonlinear systems under non-Gaussian noise and unknown process-noise statistics.

However, the memory property of fractional-order Kalman filters makes them inherently more vulnerable to outliers than their integer-order counterparts. Since FKF utilizes multiple past measurements in each iteration, any gross errors in historical data can propagate more severely through the estimation process [26]. Although robust error-state Kalman-type filters have been developed to suppress outliers and model uncertainty in attitude estimation [29], such robustness has seldom been addressed within the fractional-order framework. To maintain FKF’s high estimation accuracy while ensuring robustness, specific anti-interference treatments must be incorporated into the algorithm design.

This paper proposes a decoupled fractional-order Kalman filter (DFKF) for accelerometer-based tilt angle estimation. The principal contributions of this work are threefold: (1) A fractional-order state-space model is established for tilt angle estimation, which leverages the memory property of fractional calculus to preserve higher-fidelity system dynamics compared with conventional integer-order formulations. (2) A decoupling strategy is introduced that employs two independent state transition matrices for the state prediction and the a priori covariance computation, respectively. By incorporating a scaling tuning coefficient into the covariance prediction matrix, the prediction uncertainty is deliberately amplified, thereby enhancing the filter’s dependence on measurement updates and improving estimation accuracy under moderate noise conditions. It should be emphasised that the term “decoupling” used in this paper refers specifically to decoupling the propagation models of the state mean and of the error covariance, i.e., the first- and second-moment predictions are driven by two different transition matrices. This differs from the conventional notion of decoupling reviewed above [13,14,15,16,17,18,19], which typically separates state components, observation vectors or local estimators from one another. (3) A gross-error elimination method integrating second-order differencing with adaptive peak detection is developed to mitigate the amplified outlier sensitivity inherent in fractional-order filters arising from their memory characteristics. The effectiveness and robustness of the proposed algorithm are validated through comprehensive simulations under both noise-varying and gross-error-contaminated conditions. Compared with the previously cited fractional-order Kalman filtering approaches [20,21,22,23,24,25,26,27,28], including our earlier FKF formulations for tilt estimation and deformation monitoring [30,31], all of which share a single transition matrix between the state and covariance predictions, the added value of the present work lies in (i) explicitly decoupling the two predictions so as to correct the systematic underestimation of the prediction uncertainty that arises when no gyroscope control input is available, and (ii) equipping the fractional-order framework with a front-end gross-error pre-elimination mechanism, so that the memory property no longer propagates outliers across iterations. A detailed technical comparison is given in Section 2.3.

The remainder of this paper is organized as follows. Section 2 formulates the fractional-order state-space model, derives the fractional-order Kalman filter, and details the decoupling strategy and gross-error elimination procedure. Section 3 presents the algorithmic flowchart of the proposed DFKF. Section 4 provides simulation results and performance analysis under varying noise levels and gross-error conditions. Finally, Section 5 summarizes the conclusions and discusses future research directions.

## 2. Proposed DFKF Algorithm for Tilt Angle Estimation

### 2.1. Accelerometer-Based Tilt Angle Estimation Algorithm

The specific force output of a triaxial accelerometer is denoted as $\left(f_{x}\;f_{y}\;f_{z}\right)$. The relationship between the specific force and the gravitational acceleration in the local geographic coordinate frame is expressed as:

(1) $$\left[\begin{array}{c} f_{x}\\ f_{y}\\ f_{z} \end{array}\right]=C_{n}^{b}\left[\begin{array}{c} 0\\ 0\\ g \end{array}\right],$$

where g is the gravitational acceleration in the local geographic coordinate frame, and $C_{n}^{b}$ is the direction cosine matrix from the navigation frame to the body frame. Since the direction cosine matrix is orthogonal, $C_{n}^{b}$ is the transpose of $C_{b}^{n}$, which is defined as:

(2) $$C_{b}^{n}=\left[\begin{array}{ccc} \cos\theta \cos\psi & \sin\varphi \sin\theta \cos\psi -\cos\varphi \sin\psi & \cos\varphi \sin\theta \cos\psi +\sin\varphi \sin\psi \\ \cos\theta \sin\psi & \sin\varphi \sin\theta \sin\psi +\cos\varphi \cos\psi & \cos\varphi \sin\theta \sin\psi -\sin\varphi \cos\psi \\ -\sin\theta & \sin\varphi \cos\theta & \cos\varphi \cos\theta \end{array}\right],$$

where θ, ψ and φ denote the pitch, yaw and roll angles, respectively. Substituting Equation (2) into Equation (1) yields:

(3) $$\left[\begin{array}{c} f_{x}\\ f_{y}\\ f_{z} \end{array}\right]=\left[\begin{array}{c} -\sin\theta \\ \cos\theta \sin\varphi \\ \cos\theta \cos\varphi \end{array}\right]\cdot g,$$

From Equation (3), the accelerometer-derived roll angle $\varphi_{acc}$ and pitch angle $\theta_{acc}$ are obtained as:

(4) $$\varphi_{acc}=arctan\;\left(\frac{{\bar{f}}_{y}}{{\bar{f}}_{z}}\right),$$

(5) $$\theta_{acc}=arctan\;\left(\frac{-{\bar{f}}_{x}}{\sqrt{{\bar{f}}_{y}^{\;2}+{\bar{f}}_{z}^{\;2}}}\right),$$

where ${\bar{f}}_{x}$, ${\bar{f}}_{y}$ and ${\bar{f}}_{z}$ are the mean values of the measured specific force components. It is evident that a single accelerometer cannot resolve the heading angle. Since tilt angle estimation depends only on the roll and pitch angles, heading information is not required.

### 2.2. Fractional-Order State-Space Model

#### 2.2.1. System Dynamic Model

A brief introduction to fractional-order systems is first given for completeness. Fractional calculus generalises differentiation and integration to non-integer orders. Among its various definitions, the Grünwald–Letnikov (GL) definition is particularly suitable for digital implementation, because it expresses a fractional-order difference directly as a weighted sum of all past samples of a signal, with weights given by generalised binomial coefficients that decay gradually with the time lag. As a consequence, a fractional-order system possesses an intrinsic long-term “memory”: the current state depends not only on the immediately preceding state, as in an integer-order Markovian model, but also on the entire state history, with older states contributing progressively smaller weights. This property makes fractional-order models particularly suitable for describing slowly varying processes with long-range dependence, such as the quasi-static tilt behaviour of engineering structures.

The detailed derivation of the fractional-order dynamic system model can be found in [30,31] and is not repeated here. The resulting expressions are given as follows:

(6) Δγxk=ϕdxk−1+wk−1xk=Δγxk−∑m=1k−1mγmγmxk−m,

where γ∈(0,1] is the fractional differentiation order, $\Delta^{\gamma }$ is the fractional difference operator, $x_{k}$ is the system state vector at time step k, $\phi_{d}$ is the state transition matrix, $w_{k-1}∼N\left(0,Q\right)$ is the process noise, and γm=γγ−1⋯γ−m+1m! is the generalised binomial coefficient. The structure of Equation (6) can be interpreted as follows. The first line is the fractional-order counterpart of the conventional state equation: the γ-order difference in the state, rather than the state itself, is driven by the state transition matrix and the process noise. The second line reconstructs the state $x_{k}$ from this fractional difference, and the summation term constitutes the memory correction: each historical state $x_{k-m}$ (m = 1, …, *k*) is weighted by the generalised binomial coefficient of order *γ*, whose magnitude decays as the time lag m increases, so that recent states contribute more strongly than remote ones. When *γ* = 1, all history terms with *m* ≥ 2 vanish and Equation (6) degenerates to the standard integer-order state equation, showing that the integer-order model is a special case of the fractional-order formulation.

As discussed in Section 2.1, only the roll and pitch angles can be determined from the accelerometer specific force data. Accordingly, the state vector $x_{k}$ is defined as:

(7) $$x_{k}={\left[\varphi \;\;\delta \varphi \;\;\theta \;\;\delta \theta \right]}^{T},$$

where φ and θ are the roll and pitch angles, and δφ and δθ are their corresponding error states. The state transition matrix $\phi_{d}$ is defined as:

(8) $$\phi_{d}=\left[\begin{array}{cccc} 1 & \Delta t & 0 & 0\\ 0 & 1 & 0 & 0\\ 0 & 0 & 1 & -\Delta t\\ 0 & 0 & 0 & 1 \end{array}\right],$$

where Δt is the sampling interval of the accelerometer, which is typically set to 0.01 s.

For clarity, the general assumptions underlying the above model are summarised as follows: (1) the model is formulated directly in the discrete-time domain with a uniform sampling interval Δt, and the GL fractional difference is adopted so that no additional continuous-to-discrete conversion error is introduced; (2) the system and measurement equations are linear, which is justified because the roll and pitch angles in the considered monitoring scenarios vary within a small range; (3) the process noise and the measurement noise are mutually independent zero-mean Gaussian white-noise sequences; (4) in practical implementation, the full memory length *k* in the summation terms can be truncated to a finite window according to the short-memory principle, in order to limit the computational and storage burden; and (5) the fractional order *γ* is assumed known and constant during filtering, being selected offline according to the dynamic characteristics of the monitored process. These assumptions also delimit the applicability of the model: for strongly nonlinear or highly dynamic manoeuvring scenarios, the linear formulation would need to be extended, as discussed in Section 4.4.

#### 2.2.2. Measurement Model

The measurement model takes the tilt angles derived from the accelerometer as the observation vector:

(9) $$y_{k}=H_{k}x_{k}+v_{k},$$

where $y_{k}$ is the observation vector, $H_{k}$ is the measurement matrix, and $v_{k}$ is the measurement noise. The observation vector and measurement matrix are designed as:

(10) $$y_{k}=\left[\begin{array}{c} \varphi_{acc}\\ \theta_{acc} \end{array}\right],$$

(11) $$H_{k}=\left[\begin{array}{cccc} 1 & 0 & 0 & 0\\ 0 & 0 & 1 & 0 \end{array}\right],$$

where $\varphi_{acc}$ and $\theta_{acc}$ are the roll and pitch angles computed from the accelerometer measurements.

### 2.3. Decoupled Fractional-Order Kalman Filtering Model

In our previous work [26,30,31], a fractional-order Kalman filter was formulated by incorporating the Grünwald–Letnikov fractional-order difference operator into a linear state-space system, enabling historical state information to be retained in the prediction. The key feature of this filter lies in the historical correction terms in both the state prediction and the covariance prediction equations, which give the filter a memory property suited to dynamic systems with long-range dependence.

However, in accelerometer-based tilt angle estimation, no angular velocity measurement from a gyroscope is available as a dynamic constraint, and the state prediction relies solely on an idealised error model with limited accuracy. The conventional fractional-order Kalman filter employs a single state transition matrix $\phi_{d}$ for both the state and covariance predictions. When the state prediction is unreliable, the covariance matrix tends to underestimate the actual prediction error, causing the Kalman gain to assign excessive weight to the predicted values while underutilising the observations. To address this problem, this paper proposes a decoupled fractional-order Kalman filter (DFKF) in which the transition matrices for state prediction and covariance prediction are configured independently, allowing a more balanced weighting between predictions and observations.

#### 2.3.1. Decoupling Strategy

The central idea of DFKF is to assign independent transition matrices, $\phi_{d}^{1}$ and $\phi_{d}^{2}$, to the state prediction and the a priori covariance prediction, respectively. The state prediction retains the original transition matrix $\phi_{d}^{1}=\phi_{d}$:

(12) $$\phi_{d}^{1}=\left[\begin{array}{cccc} 1 & \Delta t & 0 & 0\\ 0 & 1 & 0 & 0\\ 0 & 0 & 1 & -\Delta t\\ 0 & 0 & 0 & 1 \end{array}\right],$$

where Δt is the sampling interval. This matrix represents a linear approximation of the tilt dynamic model based on the accelerometer error model. Since no control input $u_{k}$ is available during the prediction phase, a scaling coefficient $\alpha \;\left(\alpha \in \left(1,10\right)\right)$ is introduced to inflate the covariance prediction. The adjusted transition matrix $\phi_{d}^{2}$ for the covariance prediction takes the form:

(13) $$\phi_{d}^{2}=\left[\begin{array}{cccc} \alpha & \Delta t & 0 & 0\\ 0 & \alpha & 0 & 0\\ 0 & 0 & \alpha & -\Delta t\\ 0 & 0 & 0 & \alpha \end{array}\right],$$

where α amplifies the a priori error covariance, thereby reducing the weight assigned to the prediction in the final estimate and increasing the reliance on the observations. Based on this decoupling strategy, the state prediction equation becomes:

(14) $$\left\{\begin{array}{l} \Delta^{\Upsilon }{\hat{x}}_{k}^{-}=\phi_{d}^{1}{\hat{x}}_{k-1}^{+}\\ {\hat{x}}_{k}^{-}=\Delta^{\Upsilon }{\hat{x}}_{k}^{-}-\sum_{m=1}^{k}{\left(-1\right)}^{m}\Upsilon_{m}{\hat{x}}_{k-m}^{+} \end{array}\right.,$$

where ${\hat{x}}_{k}^{-}$ is the a priori state estimate, ${\hat{x}}_{k-1}^{+}$ is the a posteriori state estimate at the previous step, and $\Upsilon_{m}$ is the fractional-order coefficient matrix. The covariance prediction equation is:

(15) $${\hat{P}}_{k}^{-}=\left(\phi_{d}^{2}+\Upsilon_{1}\right)\;{\hat{P}}_{k-1}^{+}{\left(\phi_{d}^{2}+\Upsilon_{1}\right)}^{T}+Q_{k-1}+\sum_{j=2}^{k}\Upsilon_{j}P_{k-j}\Upsilon_{j}^{T},$$

where ${\hat{P}}_{k}^{-}$ is the a priori error covariance, ${\hat{P}}_{k-1}^{+}$ is the a posteriori error covariance at the previous step, and $Q_{k-1}$ is the process noise covariance. The use of $\phi_{d}^{2}$ in this equation inflates the prediction uncertainty, preventing excessive reliance on predicted values when reliable measurements are absent.

The use of two different transition matrices can be justified from a statistical point of view. In the standard Kalman filter, the same matrix propagates the state mean and the error covariance, because the covariance is defined as the second moment of the state prediction error under the assumption that the transition model is exact. In the present application, however, no gyroscope-derived angular rate is available as a control input, and the state prediction relies on an idealised error model of limited fidelity; the true prediction error therefore contains an unmodelled component that the nominal covariance propagation cannot capture, so the covariance computed with the single matrix $\phi_{d}$ systematically underestimates the actual prediction uncertainty. The proposed decoupling deliberately inflates the predicted covariance to account for this unmodelled uncertainty, while the state itself remains propagated by the physically meaningful matrix $\phi_{d}^{1}=\phi_{d}$ = *ϕ_d_*, so that no artificial bias is introduced into the state mean. Substituting Equation (13) into Equation (15) shows that the coefficient *α* acts multiplicatively on the a priori covariance; the strategy is thus conceptually analogous to the well-established fading-memory (covariance-inflation) technique in adaptive Kalman filtering, in which the predicted covariance is multiplied by a forgetting factor larger than one to discount an unreliable model and to increase the weight of the measurements. That is, $\phi_{d}^{2}$ does not describe the physical state dynamics, a role retained by $\phi_{d}^{1}$; instead, it serves to shape the predicted covariance so that a realistic balance between the prediction and the observations is restored. The improved accuracy observed in Section 4 under low-to-moderate noise, together with the degradation observed at high noise levels, is consistent with this interpretation.

#### 2.3.2. Outlier Rejection Strategy

Gross errors in raw accelerometer data can significantly degrade the accuracy of fractional-order Kalman filtering and may cause filter divergence. Unlike integer-order filters, the memory mechanism of fractional-order filtering repeatedly utilises data from each epoch; when an epoch contains gross errors, this repeated utilisation amplifies the resulting accuracy loss. It is therefore essential to detect and remove gross errors before applying the DFKF algorithm.

First, the accelerometer time series is inspected for anomalous pulses or discontinuities. The discrete second-order difference in the signal is computed as:

(16) $$a\prime \prime \left(t_{i}\right)=\left(a_{i+1}-2a_{i}+a_{i-1}\right)/\Delta t^{2},$$

where $a_{i}$ is the accelerometer reading at time $t_{i}$ and Δt is the sampling interval. The second-order difference amplifies anomalous features and is sensitive to gross errors. To suppress noise during detection, a moving-average filter is applied:

(17) $$\bar{a}\prime \prime \left(t_{i}\right)=\frac{1}{2M+1}\sum_{j=-M}^{M}a\prime \prime \left(t_{i+j}\right),$$

where M is the half-width of the sliding window. The filtered signal $\bar{a}\prime \prime \left(t_{i}\right)$ preserves outlier-induced deviations while suppressing high-frequency noise. An outlier is identified when:

(18) $$\left|a\prime \prime \left(t_{i}\right)\right|>\varepsilon ,$$

where ε is the detection threshold. If sample i satisfies this condition, $a_{i}$ is replaced by the mean of p valid neighbouring samples on each side:

(19) $$a_{i}^{new}=\frac{1}{2p}\left(\sum_{j=i-p}^{i-1}a_{j}+\sum_{j=i+1}^{i+p}a_{j}\right),$$

where p is the number of neighbouring samples used for interpolation. The corrected data, free from gross errors, are then used as the measurement input to prevent contamination of the state estimate by anomalous observations.

#### 2.3.3. Remarks on the Convergence of the Estimation Process

Brief remarks on the convergence of the estimation process are given here. For the fractional-order Kalman filter, Sierociuk and Dzieliński [32] derived the filter equations rigorously in the minimum-variance sense and analysed the behaviour of the estimation error, and further stability and convergence analyses of fractional-order Kalman-type filters can be found in [20,33]. Two structural properties of the proposed DFKF support convergent behaviour. First, the generalised binomial coefficients that weight the historical states decay monotonically with the time lag, so the memory terms in Equations (14) and (15) form convergent series and the contribution of remote states remains bounded. Second, the covariance inflation introduced by *α* > 1 only enlarges the predicted covariance: it keeps the a priori covariance positive definite and shifts the Kalman gain towards the measurements, so the filter tends to overestimate rather than underestimate the prediction uncertainty, which is a well-known sufficient condition for avoiding filter divergence. The bounded estimation errors and the rapid recovery after gross errors observed in all simulation scenarios in Section 4 are consistent with these properties. A rigorous convergence proof for the decoupled formulation is beyond the scope of this paper and will be addressed in future work.

## 3. Flowchart of the DFKF Algorithm for Tilt Angle Estimation the Schematic for Tilt Angle Estimation Based on the DFKF Algorithm

The DFKF algorithm consists of two stages: data preprocessing and decoupled fractional-order Kalman filtering.

In the preprocessing stage, the accelerometer time series is checked for anomalous pulses or discontinuities. When such features are found, the second-order difference in the raw signal is calculated and smoothed using a moving-average filter. Samples with filtered second-order differences exceeding a threshold ε are further checked by local extremum analysis. Identified gross errors are replaced with the mean of adjacent valid samples. The corrected data are then fed into the tilt angle calculation module, while clean data enter the module directly.

**Figure 1:**
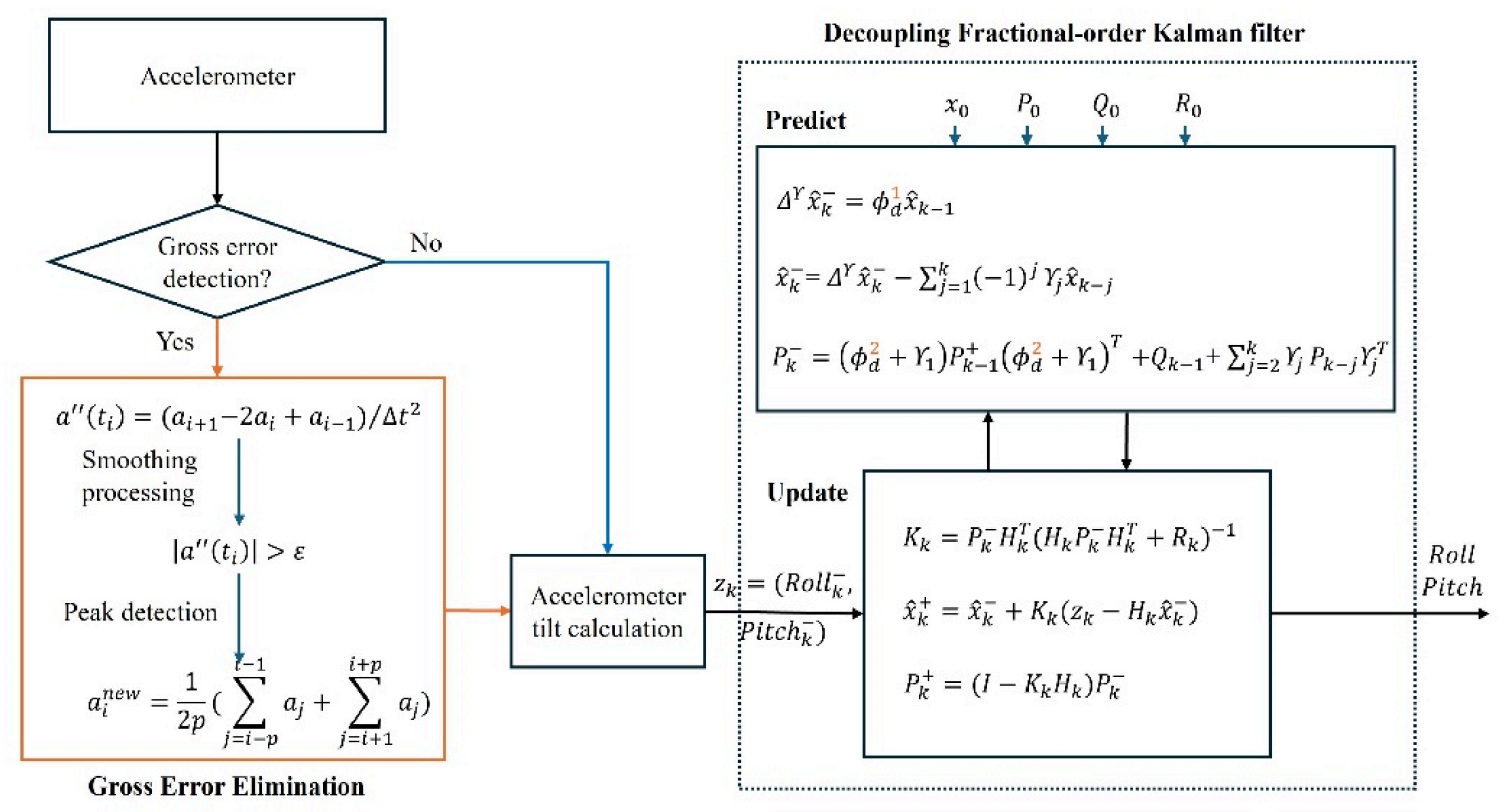
Flowchart of the DFKF algorithm for tilt angle estimation.

In the filtering stage, the state estimate ${\hat{x}}_{k}^{-}$ and the error covariance ${\hat{P}}_{k}^{-}$ are predicted using two independent transition matrices $\phi_{d}^{1}$ and $\phi_{d}^{2}$, respectively. The scaling coefficient α in $\phi_{d}^{2}$ enlarges the predicted covariance, reducing the weight of the prediction and increasing the contribution of the observations. In the update step, the Kalman gain is calculated from ${\hat{P}}_{k}^{-}$ and the measurement noise covariance, and the state estimate is corrected by the observation residual. The algorithm iterates between prediction and update to produce filtered tilt angle sequences. The flowchart of the DFKF algorithm is shown in Figure 1.

## 4. Simulation Experiment

**Figure 2:**
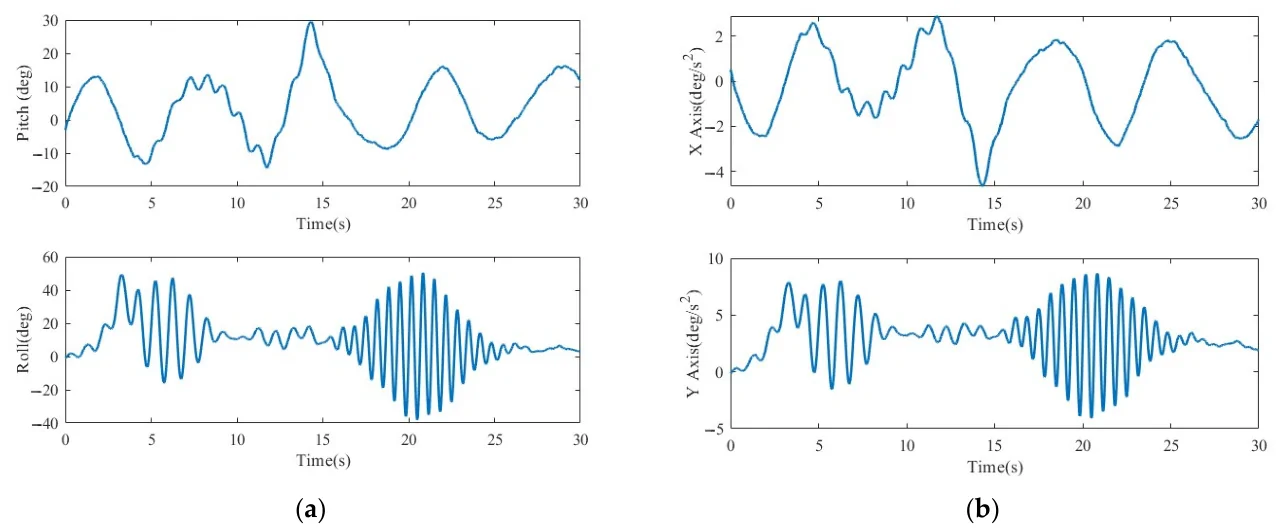
Simulation results: (**a**) tilt angles; (**b**) second-order derivatives of the tilt angles.

To validate the proposed algorithm, a 30 s tilt variation process was simulated in MATLAB R2022a. The test scenario includes both a low-dynamic component (pitch angle) and a high-dynamic component (roll angle) to assess algorithm performance under different conditions, as shown in Figure 2. Figure 2a shows the prescribed roll and pitch angle profiles, and Figure 2b shows their second-order time derivatives, which are plotted solely to visualise and compare the dynamic content of the two signals and are not used as accelerometer measurements. The corresponding noise-free accelerometer outputs, i.e., the specific-force components actually processed by the filters, were generated by substituting the prescribed roll and pitch angle profiles into the gravity-projection model of Equation (3), i.e., $f_{x}$ = −g·sinθ, $f_{y}=\;g\cdot \cos\theta \cdot \sin\varphi$ and fz = g·cosθ·cosφ with *g* = 9.8 m/s^2^, so that the simulated specific-force measurements have the correct physical units of m/s^2^ and are consistent with the measurement model of Section 2.2.2. Gaussian noise and gross errors were subsequently superimposed on these specific-force components as described below. The noisy specific-force components were then converted into the accelerometer-derived roll and pitch angles through Equations (4) and (5), forming the observation vector of Equation (10) that was processed by the three filters.

The main parameters of the simulated system and of the three filters are summarised as follows. The simulation lasts 30 s with a sampling interval of Δ*t* = 0.01 s (100 Hz sampling rate). The pitch angle follows a slowly varying (low-dynamic) profile and the roll angle a rapidly varying (high-dynamic) profile, as shown in Figure 2a, so that both quasi-static and dynamic conditions are represented in a single scenario. The state vector, transition matrix and measurement model are given by Equations (7)–(11). For a fair comparison, the process noise covariance **Q** and the measurement noise covariance R were set identically for KF, FKF and DFKF, with R chosen according to the noise level of each scenario. The fractional order of FKF and DFKF was set to *γ* = 0.9, and the scaling coefficient of DFKF was set to *α* = 2 (its selection is analysed at the end of Section 4.1). The filters were initialised with the true initial tilt angles, zero error states and a diagonal initial covariance. The parameters of the gross-error pre-elimination module were set to *M* = 5, *ε* = 0.12 and *p* = 5.

### 4.1. Performance Evaluation Under Varying Noise Levels

To evaluate the effect of noise level on algorithm performance, zero-mean Gaussian white noise with standard deviations ranging from 0 to 0.05 m/s^2^ (in increments of 0.005 m/s^2^) was added to the noise-free acceleration signal. These relatively low noise levels were adopted because the baseline signal was initially noise-free.

**Figure 3:**
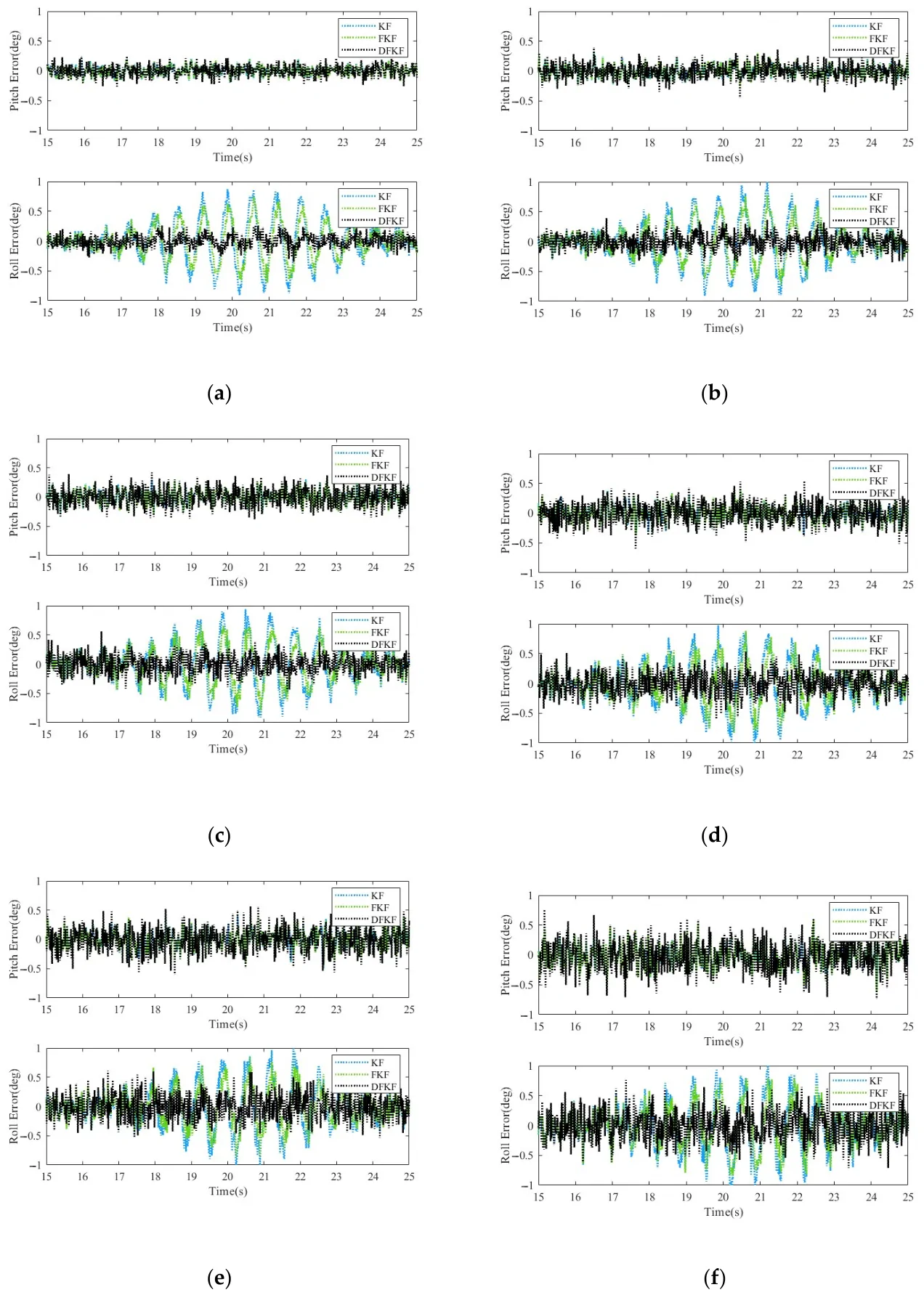

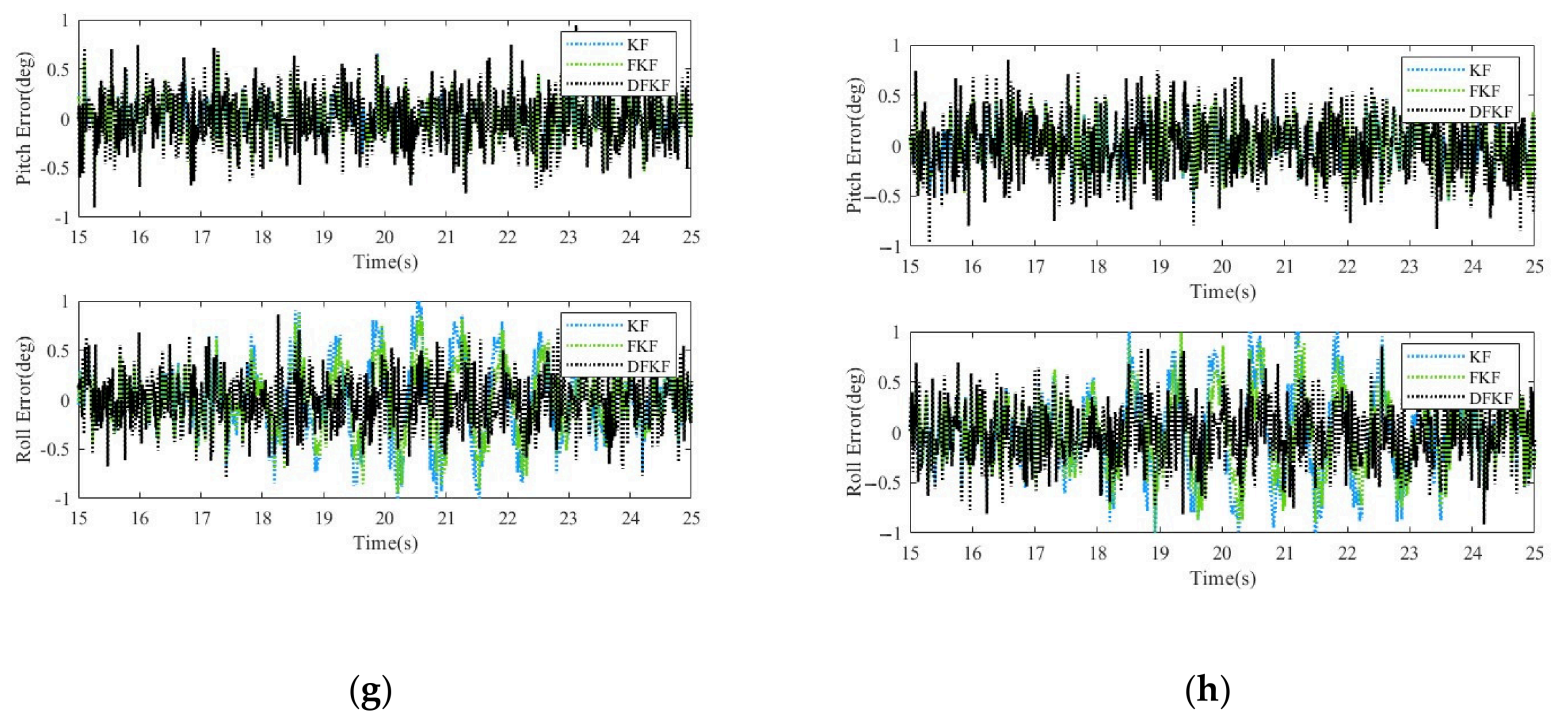
Error comparison of KF, FKF, and DFKF under different noise levels: (**a**) σ = 0.015 m/s^2^; (**b**) σ = 0.02 m/s^2^; (**c**) σ = 0.025 m/s^2^; (**d**) σ = 0.03 m/s^2^; (**e**) σ = 0.035 m/s^2^; (**f**) σ = 0.04 m/s^2^; (**g**) σ = 0.045 m/s^2^; (**h**) σ = 0.05 m/s^2^.

Figure 3 compares the estimation errors of KF, FKF and DFKF under different noise conditions, and Table 1 and Table 2 summarise the corresponding RMSE and MaxAE values. The results show that tilt angle estimation errors for both pitch and roll increase with noise level for all three algorithms. DFKF achieves the lowest mean RMSE of 0.147°, compared with 0.157° for FKF and 0.178° for KF. DFKF also yields the smallest mean MaxAE of 0.548°, compared with 0.611° for FKF and 0.726° for KF.

**Figure 4:**
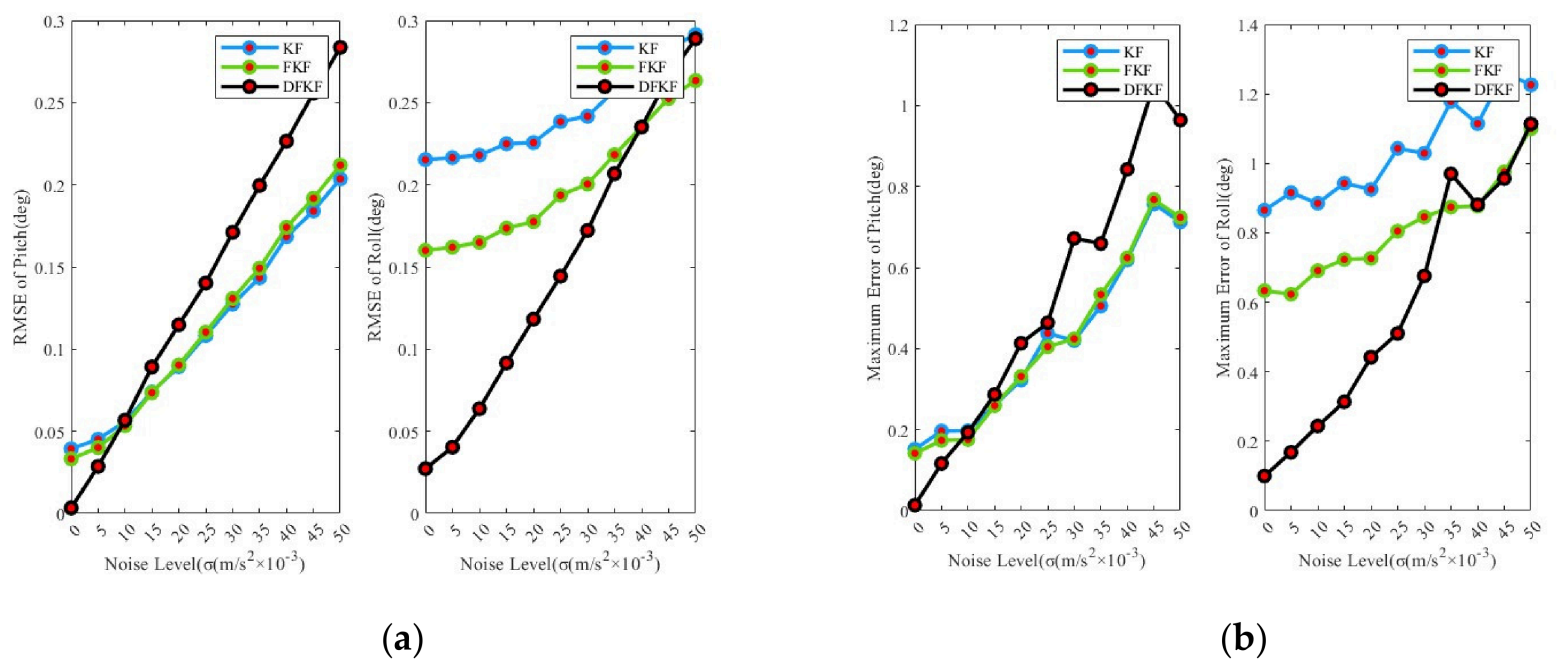
RMSE and MaxAE trends of KF, FKF, and DFKF versus noise level: (**a**) RMSE trends; (**b**) MaxAE trends.

To strengthen the statistical credibility of the results, each noise scenario in Table 1 and Table 2 and Figure 4 was repeated for *N* = 50 independent Monte Carlo runs with different noise seeds, and the values reported in Table 1 and Table 2 are the averages over these runs. For the representative case of σ = 0.02 m/s^2^, the standard deviations of the RMSE across the 50 runs were 0.004° (pitch) and 0.005° (roll) for DFKF, 0.004° and 0.006° for FKF, and 0.005° and 0.006° for KF, and the corresponding standard deviations of the MaxAE were below 0.05° for all three algorithms. These dispersions are roughly one order of magnitude smaller than the differences among the three algorithms, indicating that the reported ranking is statistically meaningful rather than an artefact of a particular noise realisation.

For a clearer comparison, Figure 4 shows the RMSE and MaxAE trends across the three algorithms as noise level increases. Figure 4a shows that DFKF exhibits the steepest RMSE increase with noise level. For pitch estimation, DFKF maintains the lowest RMSE when the noise standard deviation is below 0.01 m/s^2^, but its advantage diminishes at higher noise levels, where it is surpassed by both KF and FKF. For roll estimation, DFKF retains the lowest RMSE until the noise standard deviation exceeds 0.04 m/s^2^, beyond which FKF performs best. At 0.05 m/s^2^, the RMSE of DFKF begins to exceed that of KF.

The MaxAE trends are similar, as shown in Figure 4b. Under low-noise conditions (*σ* < 0.01 m/s^2^), DFKF achieves the lowest MaxAE for both pitch and roll. Its advantage in pitch estimation diminishes when noise exceeds 0.01 m/s^2^, while for roll estimation, DFKF and FKF show comparable MaxAE beyond 0.04 m/s^2^.

These results indicate that the error growth rate of DFKF is higher than those of KF and FKF as noise increases, with pitch estimation being more sensitive than roll estimation. This reflects a trade-off between estimation accuracy and noise sensitivity in the decoupling mechanism.

The influence of the scaling coefficient α in Equation (13) on the estimation performance was further examined through a sensitivity analysis at the representative noise level of *σ* = 0.02 m/s^2^. The value of *α* was varied over {1.0, 1.5, 2, 3, 5, 8, 10}, and the resulting mean RMSE values of DFKF (averaged over pitch and roll) were 0.134°, 0.121°, 0.117°, 0.119°, 0.125°, 0.133° and 0.138°, respectively, exhibiting a shallow U-shaped dependence. The behaviour at the two ends of the interval explains the tuning principle. When *α* → 1, the matrix ***ϕ***_d_^2^ coincides with ***ϕ***_d_^1^, the decoupling vanishes and DFKF degenerates to the conventional FKF, so α must be strictly greater than 1 for the covariance to be inflated rather than left unchanged. Conversely, as α grows large the predicted covariance is excessively inflated, the Kalman gain saturates towards the observations, and the filter gradually degenerates to a raw measurement pass-through that is fully exposed to the measurement noise; for *α* ≥ 10, the prediction weight becomes negligible and the filtering effect is essentially lost, which is why the interval (1, 10) is adopted as the practical search range. Within this interval, the performance is rather insensitive to moderate variations in α, and *α* = 2 was selected in this work as it provided the best overall balance. As discussed in Section 4.4, a larger *α* is preferable for highly dynamic signals, whereas a more conservative value suits quasi-static signals; an adaptive scheme that tunes *α* online from the innovation statistics will be investigated in future work.

### 4.2. Performance Evaluation Under Gross Errors

**Figure 5:**
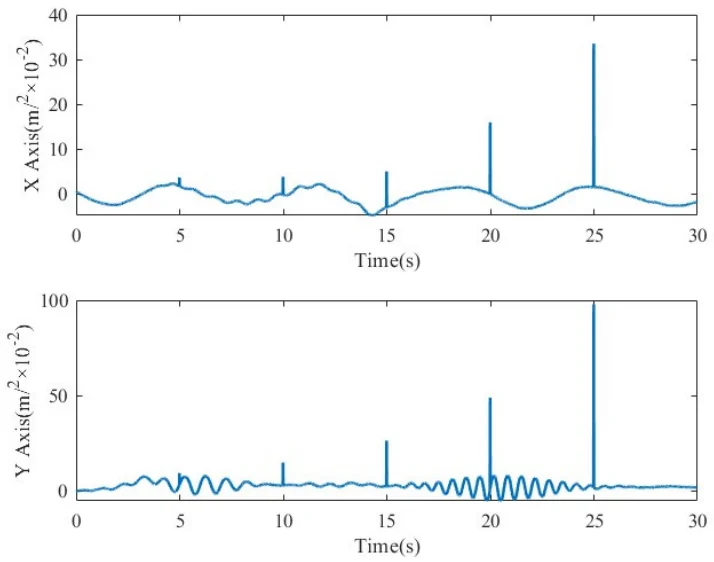
Simulation accelerations with gross errors.

To evaluate the robustness of the proposed algorithm, artificial gross errors of varying magnitudes were introduced at the 5th, 10th, 15th, 20th and 25th seconds. Gross errors of 0.01, 0.02, 0.04, 0.08 and 0.16 m/s^2^ were added to the X-axis acceleration, and corresponding errors of 0.04, 0.08, 0.16, 0.32 and 0.64 m/s^2^ were added to the Y-axis (see in Figure 5).

**Figure 6:**
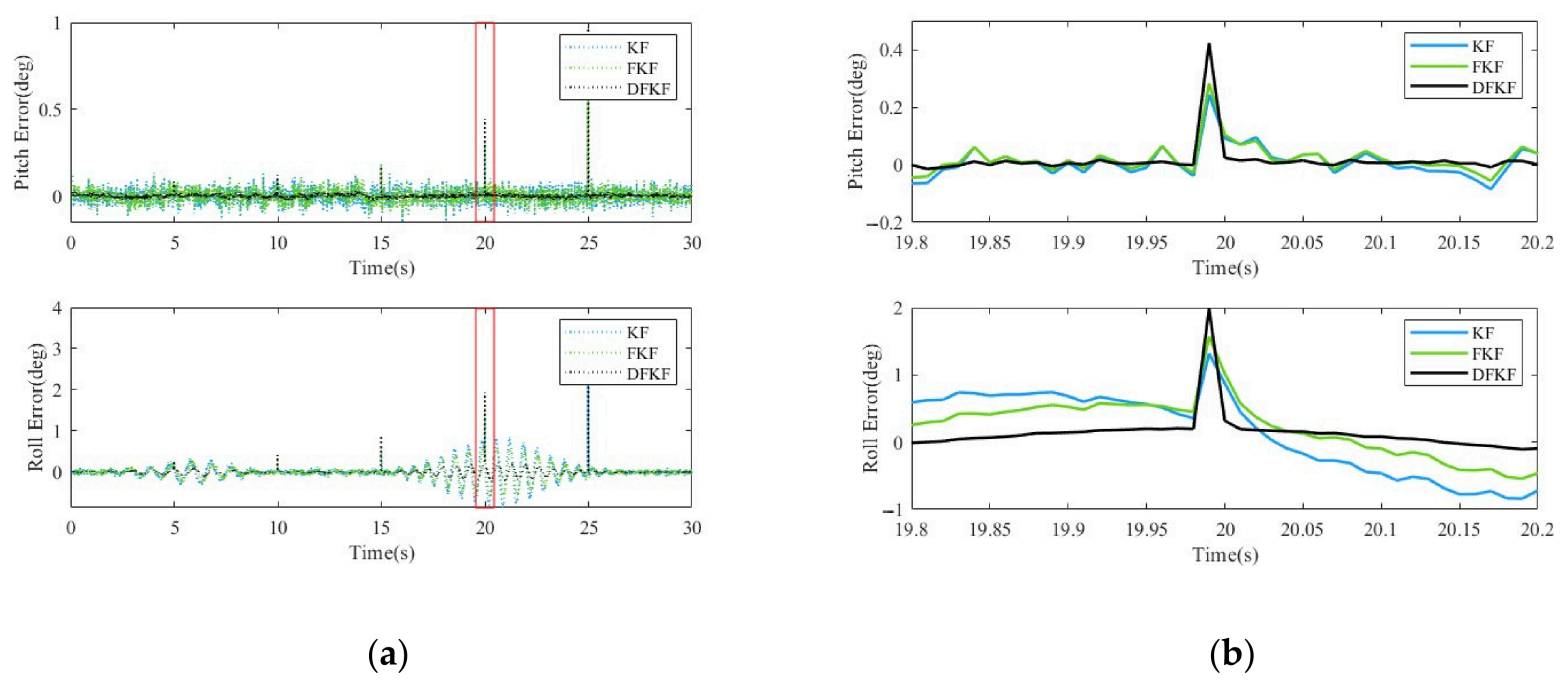
Error comparison of KF, FKF, and DFKF with gross errors: (**a**) global view; (**b**) zoomed-in view.

Figure 6 compares the error responses of the three algorithms under gross-error conditions. The red box in Figure 6a indicates the interval from 19.8 to 20.2 s, which is enlarged in Figure 6b. Figure 6b clearly reveals instantaneous error spikes coinciding with the gross-error injection epochs. Among the three algorithms, DFKF exhibits the largest error amplitude, followed by FKF and KF. However, DFKF achieves the fastest convergence, followed by FKF and KF.

For quantitative comparison, Table 3 lists the peak errors and Table 4 lists the convergence times at each gross-error epoch (5, 10, 15, 20 and 25 s). DFKF produces the largest mean peak error (0.906°), followed by FKF (0.643°) and KF (0.566°), confirming its higher instantaneous sensitivity to gross errors. However, DFKF converges in 0.012 s on average, approximately 2.8 times faster than both KF and FKF (0.034 s each). This indicates that although DFKF responds more strongly to an outlier, it recovers more rapidly once clean observations become available.

### 4.3. Gross-Error Elimination

**Figure 7:**
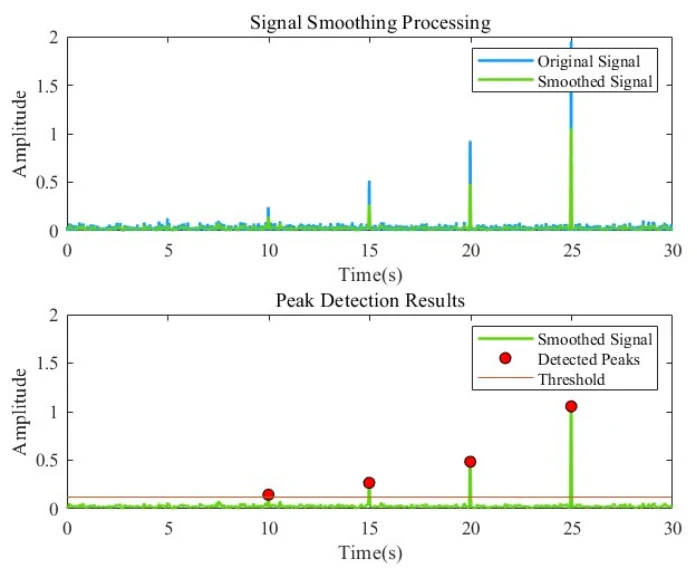
Peak detection.

The preceding analysis shows that all three filters are sensitive to gross errors. To address this, a gross-error detection method is proposed with four steps: (1) computing the second-order difference in the acceleration data; (2) smoothing the differenced signal (Figure 7, top) to suppress noise spikes; (3) setting a detection threshold (0.12 in this study); and (4) applying a peak detection algorithm for gross-error identification. As shown in Figure 7 (bottom), the method successfully detected four of the five introduced gross errors, missing only the smallest error at 5 s. Detected gross errors were replaced with the mean of adjacent valid samples.

**Figure 8:**
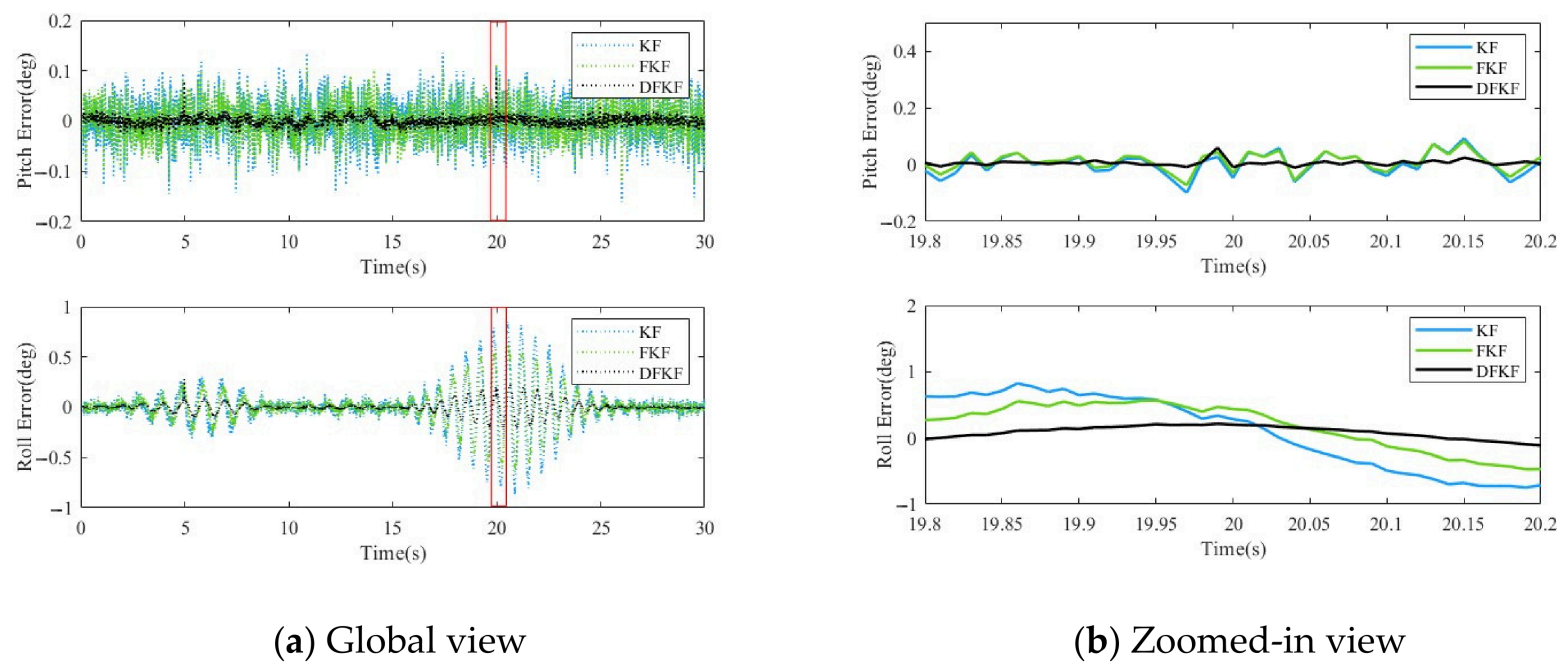
Error comparison of KF, FKF, and DFKF after gross-error elimination: (**a**) global view; (**b**) zoomed-in view.

Figure 8 compares the estimation errors of KF, FKF and DFKF after gross-error elimination. Table 5 summarises the RMSE under three conditions: error-free, gross-error-contaminated, and error-corrected.

**Figure 9:**
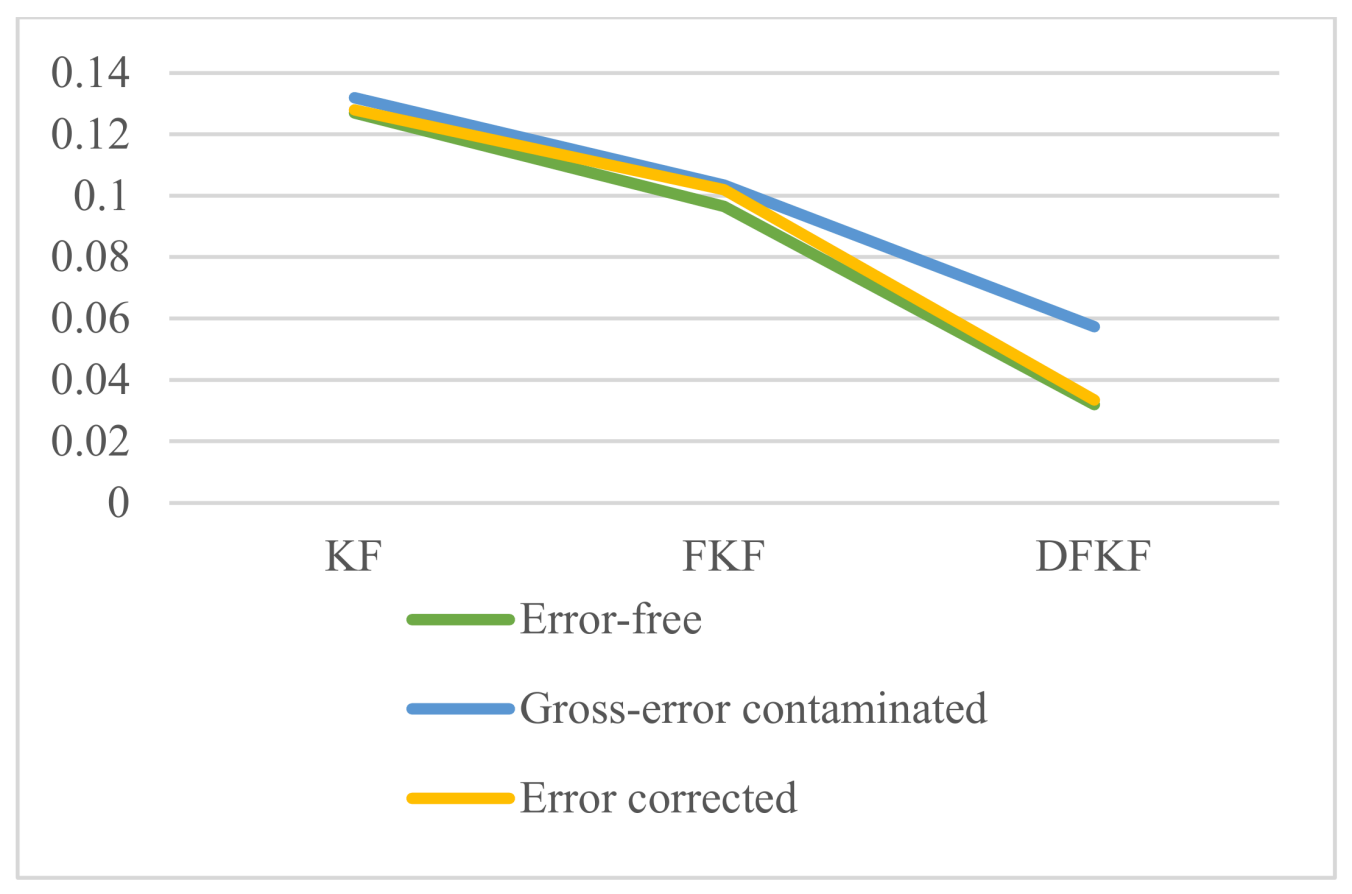
Comparison of RMSE under different conditions: clean, contaminated and processed data.

Figure 9 presents the results of Table 5 graphically. The results show that KF yields the highest RMSE across all three conditions, followed by FKF, while DFKF maintains the highest accuracy. Although gross errors degrade DFKF performance, the pre-elimination strategy restores its RMSE to near-error-free levels, confirming the effectiveness of the proposed method.

### 4.4. Discussion

In this work, we propose a decoupled fractional-order Kalman filter (DFKF) for accelerometer-based tilt angle estimation. Compared to KF and FKF, it achieves higher estimation accuracy under moderate noise conditions and faster convergence under gross-error conditions. To evaluate the proposed method, we designed simulations under two scenarios: varying noise levels and gross-error contamination.

Under noise-free conditions (Table 1, *σ* = 0), DFKF achieves markedly lower RMSE (pitch 0.003°, roll 0.027°) than KF (0.039°, 0.215°) and FKF (0.033°, 0.160°). This substantial improvement is expected because, without measurement noise, the observations faithfully represent the true tilt angles, and the decoupling strategy—which increases the Kalman gain towards the observations via the scaling coefficient *α*—fully exploits this high-quality measurement information. In contrast, KF and FKF assign a fixed portion of weight to their predictions, which, in the absence of gyroscope input, are based solely on an idealised error model and therefore less accurate than the observations themselves.

As noise increases, however, the advantage of DFKF gradually diminishes (Figure 4). For pitch estimation, DFKF maintains the lowest RMSE only when *σ* is below 0.01 m/s^2^, beyond which it is surpassed by both KF and FKF. For roll estimation, DFKF retains its advantage up to approximately 0.04 m/s^2^. We attribute this difference to the signal dynamics: the roll angle exhibits higher-frequency variation than the pitch angle in the simulation (Figure 2), meaning the state prediction model is less accurate for roll, and the increased observation weight from the decoupling compensates more effectively for this model deficiency. For the slowly varying pitch signal, the prediction model already provides a reasonable approximation, so the additional observation weight offers limited benefit but increases exposure to noise. This finding suggests that the scaling coefficient α should be selected with consideration of the signal dynamics—a larger *α* suits high-dynamic signals, while a more conservative value is preferable for quasi-static signals.

Under gross-error conditions (Figure 6, Table 3 and Table 4), DFKF exhibits the largest instantaneous peak error (mean 0.906°) compared to FKF (0.643°) and KF (0.566°), but achieves the fastest convergence (0.012 s), approximately 2.8 times faster than both KF and FKF (0.034 s). These two characteristics are closely related. From Equations (14) and (15), the decoupling strategy enlarges the predicted covariance ${\hat{P}}_{k}^{-}$, which in turn increases the Kalman gain. A larger gain means the filter responds more strongly to any single observation, including outliers, which produces a larger initial disturbance. However, the same high gain enables rapid correction at subsequent epochs when clean observations become available. KF and FKF, with their more conservative gains, are less disturbed by the outlier but require more iterations to recover. This trade-off between instantaneous sensitivity and convergence speed is an inherent property of the decoupling mechanism.

To address the outlier sensitivity, we developed a gross-error pre-elimination strategy based on second-order differencing and adaptive peak detection. As shown in Figure 7, the method successfully detected four of five artificially introduced outliers. The undetected outlier at 5 s had the smallest magnitude (0.01 m/s^2^ on the X-axis), which fell below the detection threshold ε after differencing and smoothing. Table 5 confirms that, after applying the elimination strategy, the RMSE of DFKF (pitch 0.009°, roll 0.058°) is restored to near-error-free levels (pitch 0.007°, roll 0.057°), while without elimination the contaminated RMSE (pitch 0.020°, roll 0.095°) is noticeably degraded. This result demonstrates that gross-error pre-elimination is not merely an optional preprocessing step but a necessary component when using DFKF, because the memory mechanism of fractional-order filtering (Equation (14)) would otherwise repeatedly amplify the initial large disturbance across subsequent epochs.

In conclusion, DFKF achieves the best overall accuracy among the three algorithms under moderate noise and gross-error-corrected conditions, owing to its decoupling strategy that increases the filter’s reliance on observations. While KF offers the smallest peak error under gross-error conditions and FKF provides a balanced compromise, neither matches the convergence speed or the low-noise accuracy of DFKF. The current implementation treats the scaling coefficient α as a fixed parameter; future work could develop an adaptive scheme that adjusts α based on the innovation statistics or estimated noise level, potentially combining the low-noise precision of DFKF with the high-noise robustness of conventional KF.

It should be noted that neither the decoupling strategy nor the gross-error pre-elimination procedure is restricted to the tilt-estimation model considered in this paper. The DFKF framework is applicable to the broad class of linear (or linearised) discrete-time stochastic systems in which (i) the prediction model is of limited fidelity—for example, because a control input such as a gyroscope-derived angular rate is unavailable—and (ii) the monitored process varies slowly and exhibits long-range dependence, so that the memory property of the fractional-order model is beneficial. Representative real-world applications include the inclination and deformation monitoring of high-rise buildings, bridges, transmission towers and wind-turbine towers [11,12], landslide and geotechnical subsidence monitoring, the attitude levelling of construction and agricultural machinery, robotic and self-balancing platforms, and low-cost wearable devices. For systems with pronounced nonlinearities, the framework can be naturally extended by combining the proposed decoupling with fractional-order extended or cubature Kalman filters [24,26].

A final remark concerns the order of the propagation model. The present method propagates the state and the covariance with (modified) state transition matrices, i.e., a first-order propagation, which is adequate here because the tilt dynamic model of Equation (8) is linear. For strongly nonlinear systems, however, first-order propagation may be insufficient, and higher-order approaches such as state transition tensors (STTs) offer an attractive alternative: STT-based semi-analytic filters retain the higher-order Taylor terms of the dynamics when propagating the mean and covariance [34], directional and time-varying variants of the STT substantially reduce the associated computational burden [35], and differential-algebra techniques provide an efficient implementation of such higher-order nonlinear filters [36]. Introducing an STT- or differential-algebra-based propagation into the decoupled fractional-order framework, so that the decoupling acts on higher-order moment propagation, is an interesting direction for extending DFKF to strongly nonlinear applications.

## 5. Conclusions

This paper presents a decoupled fractional-order Kalman filter (DFKF) for accelerometer-based tilt angle estimation. The method assigns two independent transition matrices to the state prediction and the covariance prediction, respectively. A scaling coefficient α is introduced into the covariance prediction matrix to enlarge the predicted uncertainty, reducing the weight of the prediction and increasing the contribution of the observations. A gross-error pre-elimination strategy based on second-order differencing and adaptive peak detection is also developed to prevent outlier propagation through the memory mechanism of fractional-order filtering.

The algorithm was evaluated under varying noise levels and gross-error conditions. Under ten noise levels ranging from 0.005 to 0.05 m/s^2^, DFKF achieved a mean RMSE of 0.147°, which is 17.4% and 6.4% lower than those of KF (0.178°) and FKF (0.157°), respectively. The mean MaxAE of DFKF (0.548°) was 24.5% lower than KF (0.726°) and 10.3% lower than FKF (0.611°). Under gross-error conditions, DFKF converged in 0.012 s on average, approximately 2.8 times faster than both KF and FKF (0.034 s). The pre-elimination strategy successfully identified four of five artificially introduced outliers and restored estimation accuracy to near-error-free levels. However, DFKF shows a higher error growth rate at elevated noise levels, particularly for pitch estimation when σ exceeds 0.01 m/s^2^, reflecting a trade-off between estimation accuracy and noise sensitivity in the decoupling mechanism.

Future work will focus on experimental validation with physical accelerometers under field conditions. Adaptive selection of the scaling coefficient α and extension of the DFKF framework to multi-sensor fusion with gyroscopes and magnetometers will also be investigated. In addition, a rigorous convergence analysis of the decoupled formulation and its extension to strongly nonlinear systems by means of higher-order state transition tensors will be explored.

## Figures and Tables

**Table 1:** RMSE of KF, FKF, and DFKF under varying noise levels (unit: deg).

σ (m/s^2^)	KF Pitch	KF Roll	FKF Pitch	FKF Roll	DFKF Pitch	DFKF Roll
0	0.039	0.215	0.033	0.160	0.003	0.027
0.005	0.045	0.217	0.040	0.162	0.029	0.040
0.010	0.056	0.218	0.053	0.165	0.057	0.064
0.015	0.074	0.225	0.074	0.174	0.089	0.092
0.020	0.089	0.226	0.090	0.178	0.115	0.118
0.025	0.108	0.238	0.110	0.194	0.140	0.144
0.030	0.127	0.242	0.131	0.200	0.171	0.172
0.035	0.143	0.257	0.149	0.218	0.200	0.207
0.040	0.168	0.272	0.174	0.235	0.227	0.235
0.045	0.184	0.283	0.192	0.253	0.256	0.266
0.050	0.204	0.291	0.212	0.263	0.284	0.289

Mean RMSE: KF = 0.178°, FKF = 0.157°, DFKF = 0.147°.

**Table 2:** MaxAE of KF, FKF, and DFKF under varying noise levels (unit: deg).

σ (m/s^2^)	KF Pitch	KF Roll	FKF Pitch	FKF Roll	DFKF Pitch	DFKF Roll
0	0.152	0.865	0.142	0.633	0.014	0.100
0.005	0.197	0.916	0.174	0.623	0.117	0.168
0.010	0.197	0.885	0.176	0.691	0.193	0.244
0.015	0.260	0.943	0.259	0.724	0.287	0.313
0.020	0.322	0.925	0.332	0.727	0.413	0.442
0.025	0.438	1.043	0.405	0.805	0.464	0.511
0.030	0.420	1.029	0.424	0.846	0.672	0.676
0.035	0.505	1.178	0.534	0.874	0.659	0.970
0.040	0.619	1.114	0.624	0.877	0.842	0.881
0.045	0.757	1.261	0.768	0.976	1.053	0.956
0.050	0.712	1.226	0.724	1.099	0.964	1.114

Mean MaxAE: KF = 0.726°, FKF = 0.611°, DFKF = 0.548°.

**Table 3:** Peak error comparison of KF, FKF, and DFKF with gross errors (unit: deg).

Epoch	KF Pitch	KF Roll	FKF Pitch	FKF Roll	DFKF Pitch	DFKF Roll
5 s	0.059	0.150	0.075	0.250	0.062	0.285
10 s	0.102	0.251	0.112	0.286	0.123	0.459
15 s	0.106	0.493	0.119	0.569	0.202	0.950
20 s	0.265	1.428	0.291	1.674	0.429	2.020
25 s	0.549	2.255	0.603	2.450	0.901	3.627

Mean peak error: KF = 0.566°, FKF = 0.643°, DFKF = 0.906°.

**Table 4:** Convergence time comparison of KF, FKF, and DFKF with gross errors (unit: s).

Epoch	KF Pitch	KF Roll	FKF Pitch	FKF Roll	DFKF Pitch	DFKF Roll
5 s	0.01	0.01	0.01	0.01	0.01	0.01
10 s	0.03	0.04	0.03	0.04	0.01	0.01
15 s	0.03	0.03	0.03	0.03	0.01	0.01
20 s	0.08	0.03	0.08	0.03	0.01	0.01
25 s	0.04	0.04	0.04	0.04	0.02	0.02

Mean convergence time: KF = 0.034 s, FKF = 0.034 s, DFKF = 0.012 s.

**Table 5:** Comparison of RMSE under different conditions: clean, contaminated and processed data (unit: deg).

Stage	KF Pitch	KF Roll	FKF Pitch	FKF Roll	DFKF Pitch	DFKF Roll
Error-free	0.039	0.215	0.033	0.160	0.007	0.057
Gross-error contaminated	0.041	0.223	0.036	0.171	0.020	0.095
Error corrected	0.040	0.216	0.034	0.161	0.009	0.058

## Data Availability

The data presented in this study are available upon request from the corresponding author.
